# Does a Five-Day Drama Program Support Men in Prison to Develop Their Self-Confidence?

**DOI:** 10.1177/0306624X231212805

**Published:** 2023-11-29

**Authors:** Emily E. L. Brooks

**Affiliations:** 1 Bournemouth University, UK

**Keywords:** prison, drama project, self-confidence, rehabilitation, substance misuse

## Abstract

This paper evaluates an established five-day drama project, designed, and delivered by a professional company, aimed to support the development of self-confidence of seven men with a history of substance misuse in a category C prison. The project involved creation of a safe space, improvised role-play, development of communication skills, and exploration of substance misuse, culminating in a performance. Audience members included prison staff, governors, healthcare staff, and prisoners. A mixed method approach was used to evaluate the project. Participant’s pre and post project self-confidence and feelings of positivity were collated by a questionnaire compromising of closed questions and measured using a Likert scale. On the last day of the project qualitative interviews were conducted using open ended questions. The findings conclude that the use of drama can support development of self-confidence in men in prison. The project encouraged skills such as, commitment, communication, collaboration, and motivation enhancing the likelihood of rehabilitation and promoting crime abstinence. Further research with a larger sample size will identify if the changes the men experienced were statistically significant and maintained.

## Introduction

The prison population in England and Wales is exponentially increasing and projected to rise to 19,944 prisoners by 2026 (Sturge & Carthew, 2022). Prisons are identified as a revolving door system where 75% of the prison population reoffend (UK Research and Innovation [UKRI], 2022). The environment tends to perpetuate criminality by entrenching disadvantage, trauma, and distress, increasing violence, relationship breakdown, addiction, self-harm, and suicide (Chamberlen et al., 2019).

The Ministry of Justice (MOJ) (2013) paper, “Transforming Rehabilitation,” aimed to enhance funding and collaboration in rehabilitation services to support offender’s access to education and restoration so they can become positive members of society. Category C prisons are promoted as institutions that aid rehabilitation as they offer opportunities to develop skills and establish employment (MOJ, 2020). However, HM Prison and Probation Service, ‘Prison Drug Strategy,’ (2019) outlines that category C prisons are least stable and report the highest rates of positive random drug testing which is linked to increased violence and crime. This links to the recent depletion in staff, safety, education, employment, health, social, and probation services has hindered the actualization and success of rehabilitation (Chamberlen et al., 2019). Arguably, more holistic, innovative, engaging projects are pivotal to enhance rehabilitation and reduce the prison population and reoffending.

Many people in prison have hindered communication skills due to poor socioeconomic backgrounds, learning difficulties, and limited education (Williams et al., 2012). To exacerbate this, the current state of education in prisons in the United Kingdom (UK) is inadequate and fails to effectively promote rehabilitation and limit reoffending (Parliament Committees 2021). Furthermore, the threat, hostility, and turbulence in prisons discourage individuals from learning rational and non-violent forms of communication (Manning, 2016). Thus, when a person in prison liaises with the judicial system or support services, which are usually populated with well-educated individuals, their ability to articulately express themselves and be understood is inadequate. Lindsay and Taylor (2018) convey that many people in prison fail to engage with programs that support their parole conditions and rehabilitation due to being unable to access or sustain the requirements. This arguably influences the cyclical notion of poor success, demotivation, and quashed self-confidence.

Abraham Maslow presented a pyramid of hierarchical needs which theorized human behavioral motivation (Maslow, 1945). To achieve self-actualization, Maslow states that a person must possess physiological wellbeing, safety, belonging and love, and social esteem. Self-actualization fosters self-confidence which is a fundamental belief in personal success, unfolding a determination to meet task demands and resilience to manage setbacks (Merriam & Grenier, 2019). Self-confidence develops in childhood from nurturing interactions and feedback from surroundings (Merriam & Grenier, 2019). Many people in prison have backgrounds consisting of trauma, violence, criminality, and antisocial behavior (Williams et al., 2012). This means that many people in prison are void of elements in Maslow’s hierarchy of needs and subsequently have stifled self-confidence. Prolonged, diminished self-confidence can generate poor mental health, perpetuated sadness, anger, and low self-worth (Merriam & Grenier, 2019). This can invoke poor coping skills, lack of motivation, anti-social behavior, and increased likelihood of suicide (Gooding et al., 2015). Thus, the negative environment in prison can hinder a person’s ability to establish self-confidence, convalescence, and transcendence, limiting likelihood of rehabilitation.

The National Institute for Health and Care Excellence (NICE) guidelines (2020) and (2019) identify therapeutic interventions for rehabilitation such as, psychosocial interventions for people with substance misuse issues and dramatherapy for people experiencing psychosis. Dramatherapy roots in psychotherapy and theatre to foster emotional and psychological growth by using dramatic play, role-play, psychodrama, and dramatic ritual to bridge the gap between a person’s limitations and aspirations (Leeder & Wimmer, 2007). The goals and theories of dramatherapy align closely with the drama project in this study by using techniques such as role-play and improvisation to challenge comfort zones, explore thoughts, emotions, experiences, and embody alternative perspectives (Feniger-Schaal & Koren-Karie, 2021). Key foundational attributes to support successful rehabilitation for those in prison.

In prisons, dramatherapy and drama programs are increasing in success and recognition at promoting rehabilitation and reducing re-offending by addressing behavioral change and enhancing therapeutic, educational, and cultural improvement agendas (Davey et al., 2015). Koch et al., (2015) add that dramatherapy based initiatives increase prisoners’ body awareness, social competence, aggression management, and positive relationships. Drama programs in prisons can offer participants a safe space to address trauma, practice healing and recovery, and establish self-worth (Afary & Fritz, 2020), which can enable a person to improve their self-confidence, social skills, and wellbeing (Balfour et al., 2019). Thus, drama in prisons is an invaluable asset to the healing journey of those in prison; the emphasis on punishment is shifted to rehabilitation, enabling positivity, motivation, and growth where behavior is explored and changed rather than perpetuated (Crawford, 2019).

Drama practice in prisons is globally identified as an agent to transform the lives of those in prisons through building community, creating social change, developing communication skills, and nurturing hope (Freebody, 2022). When a person is provided the opportunity to effectively express their thoughts and experiences, flourishment of progression, success, achievement, and self-confidence materializes (Merriam & Grenier, 2019). This was echoed by verbatim participant feedback from the project, “‘it helped me with life skills, how to talk to people properly’. . . ‘it’s not just about talking, it’s using your body to show it’. . . ‘it has helped my confidence grow’.” Therefore, drama projects that educate, encourage, and enhance communication and motivation can kindle self-confidence and equip a person with the ability and courage to embark on their rehabilitation.

This paper presents an evaluation of a drama project delivered in a prison as part of the rehabilitation and substance misuse service.

## Results

**Figure 1.**
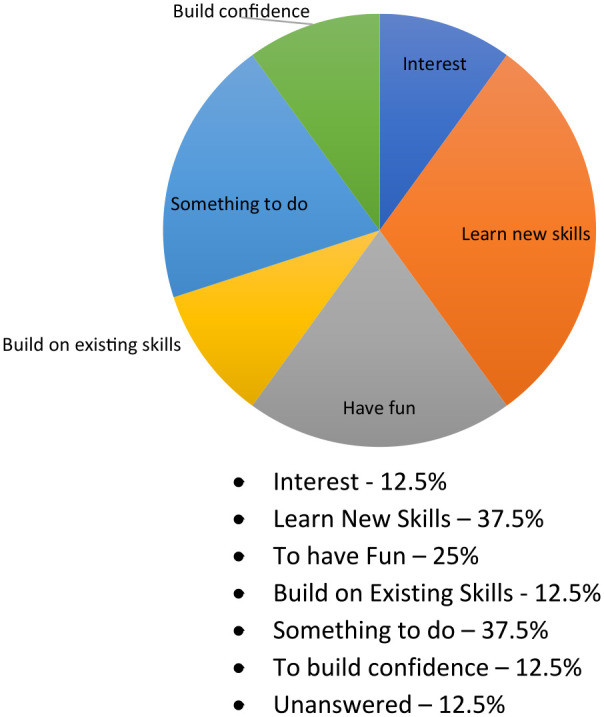
Reasons for taking part in the program (*n* = 8). Interest—12.5% Learn New Skills—37.5% To have Fun—25% Build on Existing Skills—12.5% Something to do—37.5% To build confidence—12.5% Unanswered—12.5%

Most participants indicated that they had volunteered to take part in the program to learn new skills (*n* = 3), to have fun (*n* = 2) and for something to do (*n* = 2) (Figure 1)

To note, the pre-project data shows results from eight participants and the post-project data depicts results from seven participants due to one person being unable to complete the project.

**Figure 2.**
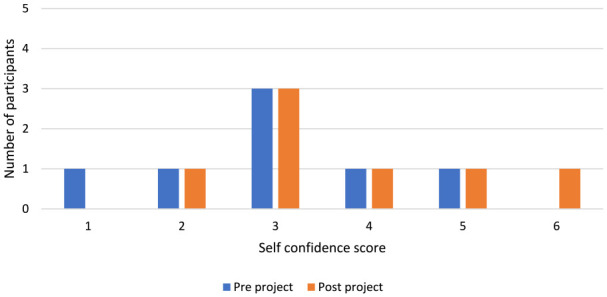
Self-confidence score of participants pre and post drama project.

Reported self-confidence for the group before the project started and on completion was similar (Figure 2) although one participant reported a big increase in self-confidence following the project.

**Figure 3.**
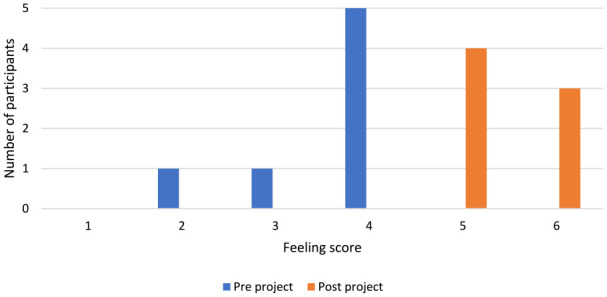
Positive Feeling score of participants pre and post drama project.

Participants reported “positive feeling” increased pre to post project (Figure 3).

### Post-project Interviews

The questionnaire responses are summarized below in Table 1. The groups’ ability to create a safe space and explore and respect each other’s ideas and experiences was reflected in the achievement of the final performance and responses to the questionnaire.

**Table 1.** Summary of questionnaire responses.

Question	Summary of key themes
1. How has being involved in the project helped you?	Increased confidence Learned more about myself Good to leave cell
2. If there was one thing you will take away from this project that you will use in your day-to-day life, what will it be and how will it help you going forward?	Awareness Confidence Social skills Dangers of drugs Trying different things
3. Can projects like this help prisoners on their rehabilitation journey?	Project can help to build confidence and skills Project could help if it was longer Project success depends on the facilitator Breaks the cycle
4. Why is it important to raise awareness about Substance Misuses through theatre?	Improves communication between staff and prisoners and organization Reminder of the dangers of drugs Using your body to show dangers Important to raise issues
5. How has this project helped your rehabilitation?	Has not helped as due release or long sentence Has helped motivation, confidence, positivity, employment potential Has reduced boredom
6. What was your favorite moment during the project?	Teamwork, seeing improved confidence, performing at the end
7. Is there anything you would change about the project?	Increased length of project Different subjects No change to project

## Method

### Evaluation Design

A single group pre-test post-test design was used to evaluate the project. On the day the project started participants were asked to complete a 6-item multiple-choice question about their motivation for participating. Participants also were asked to rate their self-confidence on the day the project started and the day the project finished using a six-point Likert scale (not good to amazing) and their feeling of positivity using a six-point Likert scale (1–6). On day 4 and 6 of the project participants completed a series of open-ended questions administered verbally. Key themes from the responses of the participants to the questions were summarized.

Convenience sampling was used to recruit participants. Eight participants volunteered and started the program. One participant decided not to continue following the start of the project and their data was excluded from the evaluation.

### Five-Day Drama Project

The drama project comprised of an established program of activities developed and delivered by a professional drama company. At the start of the project collaborative group rules were established and monitoring by the facilitators supported adherence to the rules. Day 1 and 2 involved asking participants to play a series of group games, Zap, The Name Game, Pass the Squeeze, and Guess the Mime, to build relationships, a safe-space, and trust. On day 3 participants were supported to devise an improvised role-play performance, in which, they constructed the narrative, structure, and creation of characters. The performance they constructed explored the journey of a man, called Rob, who was sentenced to prison, engaged in substance misuse, and encountered issues with debt, and prison and family relationships. The group demonstrated the Rob’s perspective and the perspectives of his fellow inmates, drug dealers, Health Services, and prison staff. The performance also included consequential thinking, exploring how changing Rob’s decisions could result in positive outcomes.

On day 5 participants performed the play to prison governors, staff, and fellow inmates. A question and answer took place after the performance, which resulted in suggestions for change in the prison to reduce drug use.

### Limitations

The number of participants was small, and the results are not generalizable. The effects of drama project were measured on the day the project completed and it is unknown if any self-reported changes were maintained. However, prior to this project and the author’s involvement, the same program was run in the same prison, by the same drama company, in the same year, to a different cohort of participants and follow up questions were asked 1 month after the project ended. Findings show participants held fond memories of the program and reported a lasting improvement on physical and mental health, self-confidence, and substance misuse abstinence and awareness.

## Discussion

The results of the evaluation suggest the drama project increased participant’s self-confidence and may have improved their rehabilitation potential.

Development of self-confidence requires the ability to subside self-doubt, mistrust, dissolution, and negative mindset (Ellis, 2013). This was supported in the project by establishing a safe space. The achievement of this was evident as participants reported feeling “good to leave the cell, able to learn more about themselves, build confidence and skills, and try different things.” This is indicative of a successful safe space because when prisoners feel safe, they are more likely to find positive trajectories and make progress (Diamond & Lanskey, 2024). In prison, a safe space is a rarity as stigma, fight for hierarchy, and fear of systematic power is rife, which can inhibit pragmatic reflection and authentic voice (DeValiant et al., 2020). Thus, a sense of security, trust, and freedom was fundamental for the participants to openly share experiences without fear of judgment or ridicule.

Improvised role-play can be an effective method when developing self-confidence (O’Toole et al., 2019). In the drama project, improvised role-play enabled the participants to experiment and share versions of reality and consider the contributors and consequences of decisions. Snyder-Young et al. (2022) states that hearing and recognizing shared experiences allows connection and collaborative, positive problem solving. Participants felt able to explore “the dangers of drugs, raise important issues, and feel better.” Key changes the participants noticed about each other was, ‘everyone was more productive and communicative. . .confidence was nice to see.’ Thus, the opportunity to improvise with thoughts and experiences arguably allowed the participants to externalize and envisage relatable situations, allowing space and opportunity to reflect and change, essential skills when building self-confidence and embarking on the journey of rehabilitation.

The drama project topic of substance misuse was chosen collaboratively through a discussion of issues that the participants were facing during their time in prison. Substance misuse, particularly psychoactive substances (PS), is rife in prison and is one of the biggest challenges in the criminal justice system, particularly in male category C prisons (HM Prison & Probation Service, 2019). Drug misuse is linked to hopelessness and helplessness and prison staff report limited treatment provision and lack of time to engage in meaningful activities (HM Prison & Probation Service et al., 2020)). Psychosocial interventions are recognized as effective treatment but are uncommon practice (NICE, 2020). Thus, implementation of dramatherapy based programs can offer multifaceted support to prisoners. Finneran and Anderson (2019) state that using drama to explore moral, political, and social issues can scaffold refined personal opinions, develop empathy, and embody alternative perspectives, which can enable self-confidence. This is supported by participant feedback as they were able to “widen their perspective.” All participants wanted to raise awareness and reduce substance misuse in prison, verbatim participant feedback added, “a lot of people are naïve to drugs and don’t see the bigger picture.” The topic choice allowed the volunteers to share common experiences and find an outlet for their concerns whilst developing personal and emotional growth.

Projects that are relevant and reflective can increase participant investment, involvement, and ownership (Armstrong et al., 2013). The participants were invested in this project, this was exampled in their common feedback about increasing the longevity of the program. Verbatim feedback added, “it was good to take part in something that’s current in everyday life.” Notably, provision of prisoner perspectives is rare because there is a perception that they are not able to offer accounts that satisfy conventional, positivistic social science (Micklethwaite & Earle, 2021). This is exampled in government funded papers (MOJ, 2013, 2020; HM Prison & Probation Service, 2019; HM Prison & Probation Service et al., 2020) as strategies to improve rehabilitation neglect the voice of people in prison. From this study, it is clear the issue of substance misuse is a shared concern for the government, public, prison staff, and prisoners. Participant feedback added, “it’s a big problem that needs sorting.” Therefore, addressing substance misuse through drama highlights the ongoing concern of substance misuse in prison, whilst advocating the prison population voice, and demonstrating that when people in prison have creative ownership over rehabilitation projects the impact and likelihood of success manifests.

Evidence of rehabilitation success is depicted in the responses from the participants. Responses stated the project, “‘helped me to come out of myself and get out of trouble, I gained employment after this’. . . ‘if I wasn’t here, I’d be sat on the wing bored doing pipes’. . . the project, ‘kept me busy and occupied my mind’.” This suggests the project was successful at offering positive and productive distraction where participants fostered improved self-confidence and ability to seek behavioural change and employment, key elements of rehabilitation. Additionally, influence on self-confidence, optimism, and motivation for rehabilitation is evident as notable feedback included, “I never get up in the morning and since I’ve been here, I’ve got up at 7 in the morning to come.” Notably, this drama project is not the solution for all issues relating to prison population low self-confidence or inadequate rehabilitation. However, there is evidence to highlight a drama project can be a positive influencer and offer development in self-confidence, motivation, and rehabilitation.

The findings of the participant’s increased self-confidence could link to a person’s relationship with substance misuse. The use of drugs such as PS, can manifest feelings of elation and gratification (Coon & Mitterer, 2012). Similarly, drama can elicit feelings of euphoria and satisfaction (Zunshine, 2014). This is echoed in the comparison of the drama project findings in Figures 2 and 3. However, substances can feign feelings of self-confidence and when drug affects diminish, a person is left depleted (Coon & Mitterer, 2012). This impact can be similar in drama performance, Dolan (2010) states that performing can offer an apotheosis unlike everyday occurrences and a subsequent comedown. Thus, drama can emulate the drug experience, but offers an outlet to achieve a natural high and intrinsic rewards, such as self-confidence, in a safe and controlled environment, whilst unearthing psychological and social needs, which are often the core of substance misuse, to manage comedowns. This is perhaps why those experiencing substance misuse are attracted to drama, as individuals can search for alternative ways to maintain the drug experience without the chemically induced high (Hanson et al., 2014). Notably, although the performance at the end was the groups biggest worry it was also the most reported favorite part of the project. Alongside this, all participants remained drug free for the entirety of the program, one participant stated the project helped because, “I’m ready to come out, I’m ready to be a member of the public and fit in with the social norm.” Evidently, drama projects in prisons can offer behavioral change but perhaps they also attract those experiencing substance misuse, who are arguably individuals most difficult to reach and in need of rehabilitation support.

Notably, substance misuse, drama creation, and performance come to an end and can lose affect overtime. The participant’s post-project feedback was collated after the performance, and their feeling of positivity significantly increased, evidenced in Figure 3. However, rapid collation means there is possibility the participant’s elation may be apparent and not yet diminished. For example, the comedown after performing can leave lingering emotions, excess adrenaline, and visceral needs, such as thirst, hunger, exhaustion, or pain, contributing to a disruption in behavior and decision making (Panoutsos, 2021). After the performance the participants re-joined the typical prison regime and became resubmerged in prison happenings, possibly meaning they were vulnerable to reverting to ingrained maladaptive coping mechanisms, such as substance misuse. A possible solution to this could involve the education system in prison embedding alternative subjects such as drama into their curriculum that could run consistently. Although, as previously discussed, U.K. education in prisons is inadequately delivering of core subjects or effectively supporting rehabilitation.

The lasting impact of positivity from this project is unknown, yet there is evidence of a cyclical link between the participant’s feelings of positivity and self-confidence. This notion transpired in participant feedback stating the project, “breaks the cycle.” When an individual feels jubilant their likelihood of rehabilitation increases because broader, flexible, creative thinking unfolds, which influences informed choices and initiates action and long-lasting adaptive consequences (France, 2015). Positivity and self-confidence are cyclical and can develop into a self-sustaining cycle (Malik, 2022). Thus, participant exposure to positivity and self-confidence development could provide enough motivation to break maladaptive coping mechanisms and maintain rehabilitation engagement.

Plausibly, the participants in this project possessed a degree of self-confidence to volunteer. This is supported by most of the participants demonstrating a motivational reason to complete the project such as, “to learn new skills, to have fun, and to build confidence.” Drama can be a daunting prospect, particularly for those in prison who arguably lack self-confidence, positivity, and autonomy. Thus, drama projects may exclude those who are void of self-confidence and at most risk of perpetuated criminality and suicide. Additionally, a person in prison may view drama projects as a frivolous activity when adjacent to prison concerns or parole/sentence/release conditions.

Numerous people in prison are under threat, victims of bullying, lack supportive relationships, and experience isolation (Chamberlen et al., 2019). Williams et al. (2012) state that these issues continue after release; for many, community support is required, which is often unsatisfactory, meaning most fall back into crime and hardship. A Ministry of Justice Study (MOJ) (2013) found a strong correlation between homelessness, illicit drug use, hazardous drinking, and people released from prison on community orders. Approved Premises (AP) are designed to provide people released from prison a supportive and structured environment to manage risk and reduce further offending; however, these establishments often house those who are unemployed, experiencing poor mental health, and/or involved in criminal behavior such as substance misuse (Prisons & Probations Ombudsman, 2021). This suggests that individuals identified as requiring support and supervision in the community are intrenched in poor environments that perpetuate criminality and hinder rehabilitation. Findings from this study reflected corresponding experiences in relation to substance misuse, with one participant stating, “substances can dull your voice and you suffer in silence.” Therefore, many in prison have grim and pessimistic experiences and outlooks which can ridicule the prospect of engaging in drama to support their situation.

Nevertheless, this project succeeded in recruiting and maintaining seven participants, all of whom had differing motivations to partake and were experiencing prison and outside life challenges. This is perhaps exampled by participants feedback that evidenced motivation to stay despite reservations, “the project has not helped due to upcoming release or a long sentence,” yet, they remained present for the project entirety. Additionally, encouraging reluctant people to engage can derive through word of mouth and promotion. This is exampled by a participant who stated, “before I wouldn’t try this. . . I will get involved more because I’m normally quite shy, now I feel more confident.” Thus, if drama projects can reach a proportion of those in prison and develop their self-confidence, it is progress and may enable progressive rehabilitation engagement.

This project did not explore longer project delivery timeframes and if this would enhance or hinder participant self-confidence, positivity, and lasting impact on rehabilitation. Longer delivery could diminish the project’s novelty and success. For example, practicalities of achieving sustained funding, attendance, and commitment from sponsors, staff, and participants could hinder the impact of the project. However, most of the participants wanted the project to be longer, which is an attribute to the project’s success. A longer project could enhance and sustain a participant’s self-confidence, which may be prevalent for individuals serving longer sentences. Verbatim feedback from the interviews highlighted, “I have two years left and that’s a long time to be sat on the wing, and you forget things.” Additionally, longer projects may entice some hard-to-reach participants as increased exposure may allow individuals more time to consider partaking. Alongside this, more time may enable additional prisoner participant roles, such as set designers, script writers, and directors, which would broaden the project and enable individual’s increased and alternative opportunities to be involved.

Overall, the evaluation of the drama project suggests the use of drama programs can support men in prison to develop their self-confidence and improve rehabilitation potential. This is evidenced and discussed in the comparative quantitative data and qualitative feedback comments. Notably, further research is needed to identify if the changes are maintained and significant.
